# Declining atmospheric deposition of heavy metals over the last three decades is reflected in soil and foliage of 97 beech (*Fagus sylvatica*) stands in the Vienna Woods☆

**DOI:** 10.1016/j.envpol.2017.06.080

**Published:** 2017-07-11

**Authors:** Selina Türtscher, Pétra Berger, Leopold Lindebner, Torsten W. Berger

**Affiliations:** Department of Forest- and Soil Sciences, Institute of Forest Ecology, University of Natural Resources and Live Sciences (BOKU), Peter Jordan-Straße 82, 1190 Vienna, Austria

**Keywords:** *Fagus sylvatica*, Heavy metals, Long-term trend, Plant micro-nutrition, Stemflow

## Abstract

Rigorous studies on long-term changes of heavy metal distribution in forest soils since the implementation of emission controls are rare. Hence, we resampled 97 old-growth beech stands in the Vienna Woods. This study exploits an extensive data set of soil (infiltration zone of stemflow and between trees area) and foliar chemistry from three decades ago. It was hypothesized that declining deposition of heavy metals is reflected in soil and foliar total contents of Pb, Cu, Zn, Ni, Mn and Fe. Mean soil contents of Pb in the stemflow area declined at the highest rate from 223 to 50 mg kg^−1^ within the last three decades. Soil contents of Pb and Ni decreased significantly both in the stemflow area and the between trees area down to 80–90 cm soil depth from 1984 to 2012. Top soil (0–5 cm) accumulation and simultaneous loss in the lower soil over time for the plant micro nutrients Cu and Zn are suggested to be caused by plant uptake from deep horizons. Reduced soil leaching, due to a mean soil pH (H_2_O) increase from 4.3 to 4.9, and increased plant cycling are put forward to explain the significant increase of total Mn contents in the infiltration zone of beech stemflow. Top soil Pb contents in the stemflow area presently exceed the critical value at which toxicity symptoms may occur at numerous sites. Mean foliar contents of all six studied heavy metals decreased within the last three decades, but plant supply with the micro nutrients Cu, Zn, Mn and Fe is still in the optimum range for beech trees. It is concluded that heavy metal pollution is not critical for the studied beech stands any longer.

## 1 Introduction

Anthropogenic emissions of air pollutants and subsequent deposition of heavy metals are known to cause negative effects on chemical and biological processes in soils. Especially, in the northern hemisphere, large areas were polluted by various heavy metals via atmospheric deposition from industrial and traffic emission sources including power generation (Bergkvist et al., 1989; Steinnes and Friedland, 2006). Long-term acidification of terrestrial ecosystems caused a reduction of cation exchange capacity and increasing mobilization of heavy metals in soils with decreasing pH (Blake et al., 1999; McKenzie, 1980). Ecotoxicological risks associated with elevated heavy metal contents in soils include reduced development of roots and shoots of plants, decline of biomass production, reduced abundance of soil fauna and decreased nutrient contents of soils and foliar tissues (Balsberg-Påhlsson, 1989; Menon et al., 2007; Rautio et al., 2005). High atmospheric inputs to forest ecosystems caused increasing plant uptake rates of the relatively mobile heavy metals zinc (Zn) and manganese (Mn) and top soil accumulation of the less mobile metals copper (Cu) and lead (Pb) (Friedland, 1992; Siccama and Smith, 1978). Though atmospheric deposition of these heavy metals decreased in many regions, current releases via mobilization of this legacy may still represent a potential danger for forest ecosystems (Friedland, 1992).

During the past decades much efforts have been done to reduce emissions of Pb and other heavy metals. Emission control legislations improved industrial cleaning techniques and phasing out of leaded petrol showed significant positive effects. In Austria, leaded petrol for automobiles was finally prohibited in 1993. By implementing the Protocol on Heavy Metals to the Convention of Long-Range Transboundary Air Pollution (CLRTAP) emissions of heavy metals declined markedly in Europe, raising the question of metal behavior under conditions of decreasing inputs. E.g., in Europe, emissions of heavy metals decreased from 1990 to 2012 by 89% for Pb, 67% for nickel (Ni), 42% for Zn and 1% for copper (Cu; EEA, 2014). In Austria, emissions of Pb declined from 215 t (1990) to 15 t (2014) by 93% (Umweltbundesamt, 2016). Moss surveys, conducted in Austria, showed a decline of Cu, Zn and Ni contents by 7%, 22% and 51%, respectively, from 1991 to 2005 (Zechmeister et al., 2008). The topic is politically important because billions have been invested in cleaning up the emissions that cause Acid Rain and associated heavy metal deposition. So it is worthwhile to know how much improvement has been achieved. In order to assess the achievements of environmental protection measures, repeated soil sampling is a good way to record changes of soil properties over time. In general, the residence time of elements in soils is longer than in other environmental media. As a consequence, despite successful reductions of heavy metal emissions, the impact of these metals may remain a major concern (Desaules et al., 2010; Johnson et al., 1995; Miller and Friedland, 1994). There are several reports suggesting that the residence time of Pb in forest soils could be in the order of several hundred years (Friedland and Johnson, 1985; Turner et al., 1985). However, more recent studies showed that Pb movement down the soil profile may be more rapid than previously thought and contamination of groundwater could become an issue (Friedland et al., 1992; Johnson and Richter, 2010; Miller and Friedland, 1994). Therefore, this topic is scientifically important as well.

Forest ecosystems are sensitive to atmospheric pollution because of high deposition loads due to the interception capacity of forest canopies (Kobler et al., 2010). Stemflow of beech (*Fagus sylvatica*) represents a high input of water and elements (Chang and Matzner, 2000), which is why deposition of acidifying substances may be significantly higher close to the stem compared to areas affected by throughfall only (Kazda et al., 1986; Matschonat and Falkengren-Grerup, 2000). As a consequence, microsites affected by stemflow of beech were severely acidified and enriched with heavy metals due to the interception of atmospheric pollutants via the canopy (Bini and Bresolin, 1998; Fantozzi et al., 2013; Kazda, 1983; Rampazzo and Blum, 1992; Skrivan et al., 1995; Wilcke et al., 1998). Comparison between chemical parameters of Austrian soils from the infiltration zone of stemflow near the base of the stem and from the between trees area by Lindebner (1990; sample collection in 1984) proved a significant impact of atmospheric pollutants: soil acidification, loss of base cations and heavy metal accumulation. Focusing on the spatial heterogeneity of soil chemistry related to the distance from beech stems enables the study of recovery of differently polluted soil within the same stand, since the infiltration zone of beech stemflow received much higher deposition loads than the reference area between the trees in the past.

Responses to environmental pollution can be studied in different environmental media. High contents of Cd and Zn in foliage of plants on heavy metal contaminated soils were reported by Brekken and Steinnes (2004) and Ohlson and Staaland (2001). A biomonitoring program in Germany revealed a significant decline of Pb contents in beech foliage over the past two decades. It is concluded that heavy metal contents in the foliage of forest trees can be used as indicators of pollution. There are many studies reporting the usefulness of plants as bioindicators for assessing temporal changes of the environmental quality (Amores and Santamaría, 2003; Brekken and Steinnes, 2004; Franzaring et al., 2010).

During the last decades many studies investigated heavy metal contents in cultivated soils (e.g., Agbenin and Felix-Henningsen, 2001) and urban soils (e.g., Biasioli et al., 2006; Imperato et al., 2003; Luo et al., 2008) but information on heavy metal contamination in forest soils is scarce. To our knowledge, there is no literature documenting changes of heavy metal contents in forest soils over a time span of several decades. Hence, we resampled 97 of 152 old-growth beech stands in the Vienna Woods, documented by Lindebner (1990) in the early 1980s. This study exploits an extensive data set of soil (infiltration zone of stemflow and between trees area) and foliar chemistry from three decades ago, and thus represents an opportunity that may be unique worldwide. We recently published part one of the overall study, focusing on soil acidification and macro nutrition of these beech stands (Berger et al., 2016). The biogeochemistry of heavy metals in these forest ecosystems, however, is the issue of the current second part of the same overall study. We hypothesized that declining atmospheric deposition of heavy metals is reflected in soil and foliar chemistry and addressed the following questions:

1) Have soil contents of Pb, Zn, Cu, Ni, Mn and Fe changed as atmospheric heavy metal deposition declined over the last three decades?
2) Are heavy metal soil contents related to forest site factors (geography, geology, terrain, aspect)?
3) Are beech (*Fagus sylvatica*) foliar contents useful as bioindicators for heavy metal contamination of soils?

## 2 Materials and methods

### 2.1 Study area and study sites

**Fig. 1:**
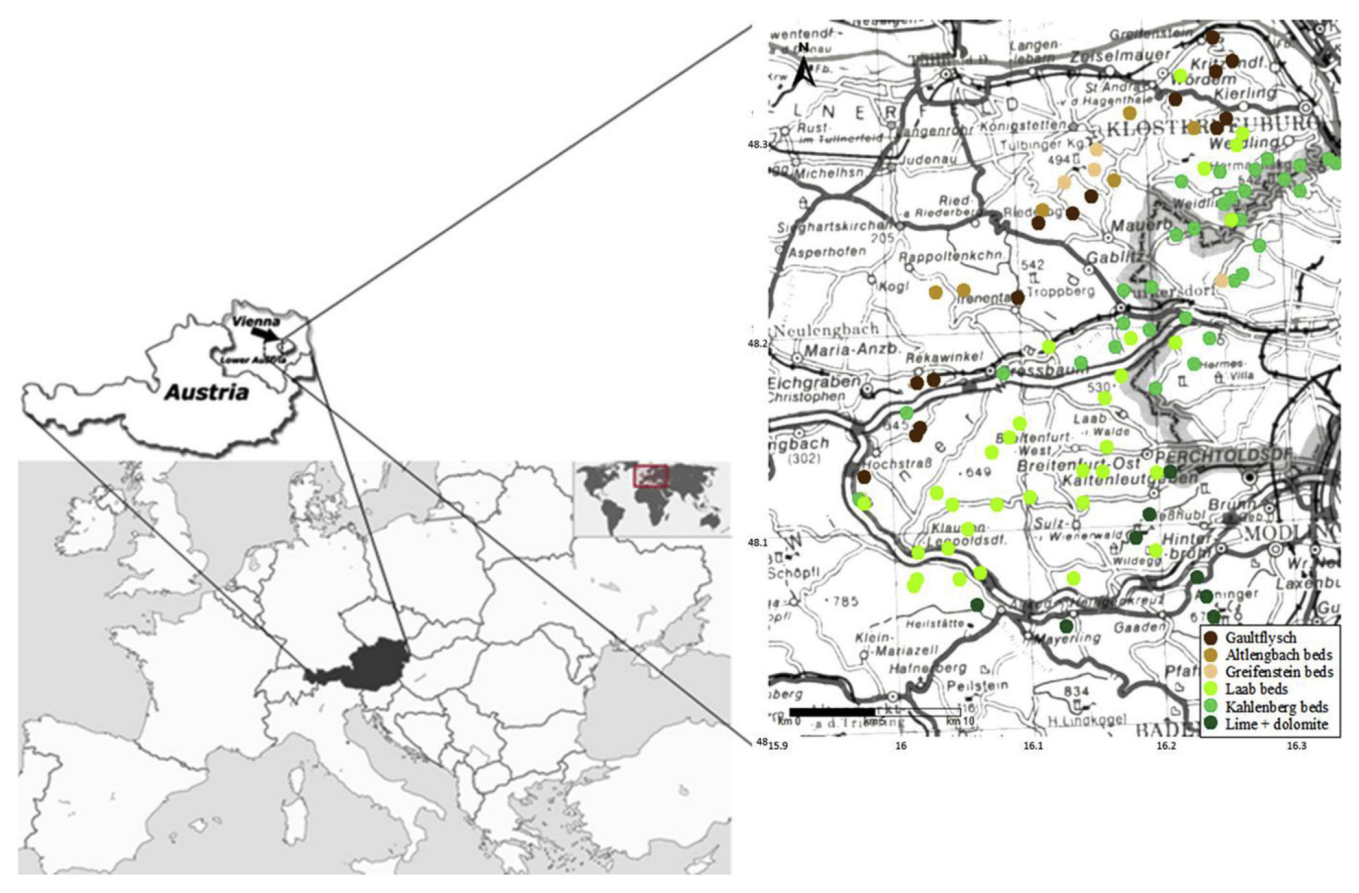
Location of 97 study sites in the Vienna Woods in 2012 and their classification according to the geological parent material for soil formation.

We selected 97 beech stands throughout the Vienna Woods, a forested highland that forms the foothills of the Northern Limestone Alps in the federal states of Lower Austria and Vienna (Austria, Fig. 1). The total area is about 125.000 ha, situated north, west and south of the City of Vienna. Geologically, the Vienna Woods can be divided into north and south. The bedrock of the northern, major part of the Vienna Woods is Flysch. The Flysch zone is a narrow strip in the foothills of the Northern Limestone Alps from west to east throughout Austria and consists mainly of old tertiary and mesozoic sandstones and clayey marls. The southern, much smaller part of the Vienna Woods is limestone. Beech (*Fagus sylvatica*) is the main tree species in the Vienna Woods, representing 50% of the standing timber volume. Other species like oak (*Quercus sp.*), black pine (*Pinus nigra*) and Norway spruce (*Picea abies*) make up a relatively small percentage of the forest cover (Plöchinger and Prey, 1974; Rieder, 2002).

The Vienna Woods are located at the transition between two climate zones, the moderate central European transitional climate and the dryer Pannonian climate. There are two main wind directions, west winds all over the year and south-east winds mostly in fall and winter. The mean annual precipitation varies between 600 and 900 mm and the mean annual temperature is 8–9 °C. Elevations range from about 180 m to over 800 m a.s.l. (Plöchinger and Prey, 1974; Rieder, 2002).

All soils on Flysch were classified as pseudogley (Scheffer and Schachtschabel, 2010; WRB classification: endostagnic cambisol), since horizons with a high fraction of fine material (loam to clay) cause temporary waterlogging (stagnation zone at approximately 40–50 cm soil depth). Soils on limestone (only 8 out of all 97 sites) were classified as Kalkbraunlehm (Scheffer and Schachtschabel, 2010; WRB classification; endoleptic cambisol). The prevalent humus forms are mull and intermediate types between mull and moder. The nutrient release of both bedrocks is high, indicating quick turnover of the forest litter layer and nutrient rich soils.

In the early 1980s, 152 pure old-growth beech stands were selected. At that time, all stands were older than 80 years and had a stand density index of ≥ 0.8. More details about the sites are given by Lindebner (1990). Three decades later, 97 of the 152 beech stands still existed for repeated sampling. The rest of the sites had been cut. The selected 97 study sites were not thinned between 1984 and 2012. Based upon our field work and geological mapping (e.g., Plöchinger and Prey, 1974) we assigned each stand either to lime and dolomite or to one of the following geologic parent materials on Flysch: Gaultflysch, Altlengbach beds, Greifenstein beds, Laab beds, Kahlenberg beds (Fig. 1). The geographical distribution of the stands and their parameters were assessed via surface mapping. Each forest stand was allocated to the following terrain classification system: lower-, middle-, upper slope and hilltop as well as aspect (N through NE, E to NW).

### 2.2 Soil sampling and analysis

The soil sampling procedure is given by Berger et al. (2016) but bears repeating at this point. In summer 1984, top mineral soil samples (0–5 cm depth) from the infiltration zone of stemflow (20 cm downhill from the base of the stem, S 0–5) and from the between trees area (at least 3 m away from beech stems, B 0–5) were taken with a cylinder (diameter 50 mm; height 50 mm, inserted from the top). In each case, there were seven distributed replicate samples (1 sample per beech tree) at each stand which were pooled before chemical analysis. One year later (summer 1985), soil samples were taken with a soil auger (diameter 20 mm, half open steel pipe) from 30 to 40 cm (B 30–40) and 80–90 cm (B 80–90) soil depth in the between trees area. In this case, 4 replications per stand were pooled before analysis. Assuming that the deep soil parameters did not change within one year, we refer all these samples to the year 1984 throughout this paper.

In spring 2012, soil samples were collected using the same method used by Lindebner (1990) in 1984. Additionally, we took soil samples from 10 to 20 cm soil depth from the infiltration zone of stemflow (S 10–20) and the between trees area (B 10–20). The 20 mm diameter auger was used to take 7 soil samples from the stemflow area (S 10–20) adjacent to the 0–5 cm collections which were pooled before analysis. In the between trees area the identical 4 sub-sites were used for B 10–20, B 30–40 and B 80–90.

Soil chemical analyses were carried out following routine procedures as suggested by Blum et al. (1989) for the standardization of Austrian soil surveys. Mineral soil samples were thoroughly homogenized and passed through a 2 mm sieve. Soil pH and C_org_ was measured by Berger et al. (2016) in the identical soil samples. Soil acidity was measured as pH with a glass Ag/AgCl combination electrode with KCl reference electrode: 10 g soil was mixed with deionized H_2_O, stirred, and the pH was measured next morning 30 min after stirring again (ÖNORM L1083). Organic C was calculated total C (Wösthoff Carmhomat ADG 8, Germany, ÖNORM L1080) minus C_CaCO3_, that is, reaction of carbonates with HCl (volumetric determination of emerging CO_2_ according to ÖNORM L1084 after Scheibler).

Mineral soil was analyzed for total contents of Pb, Cu, Zn, Ni, Mn and Fe. In 1984, total heavy metal content was determined by acid digestion using nitric acid (65% HNO_3_). One gram of soil sample was placed in a 75 ml digestion tube (Kjedahl flask) and 10 ml nitric acid (65% HNO_3_) was added. The sample was heated for 30 min at 60 °C, and then the temperature was increased to 140 °C at which the sample was boiled for 1 h. After cooling, 20 ml of distilled H_2_O was added to the sample and heated up again. Thereafter, distilled water was added again and the solution was filtered into a 50 ml flask. The detailed method is given by Lindebner (1990), who decided to use this easy to handle nitric acid digestion instead of the common aqua regia digestion after proving for a set of samples that both methods revealed identical results. In 2012, an aqua regia (HCl/HNO_3_; ÖNORM EN 16174) digestion was used to determine total heavy metal contents (2 g soil : 15 ml HCl : 5 ml HNO_3_). Lead, Cu, Zn, Ni, Mn and Fe analyses were conducted by graphite furnace atomic absorption spectrometry (GF-AAS, Perkin Elmer 3030, USA) in 1984 and by inductive coupled optical emission spectrometry (ICP-OES, Optima 3000 XL, Perkin Elmer, USA) in 2012. All methods were run parallel for several years, yielding comparable measurements.

### 2.3 Foliar sampling and analysis

The same leaf samples which we analyzed in part one of the overall study for macro nutrients (see Berger et al., 2016) were now analyzed for heavy metal contents. In late August/early September 1984 and in early September 2012 leaf samples of beech were collected with a shot gun from the upper crown of two to three trees per site. All subsamples per site were pooled before analysis, yielding approximately 60–100 leaves. To avoid contamination only intact leaves without any shot through, attached to a struck branch, were collected. Foliage samples were dried at 105 °C and ground. Lead, Cu, Zn, Ni (only in 2012), Mn and Fe were measured as total contents after digestion with HNO_3_/HClO_4_ (ÖNORM L1085) by GF-AAS in the 1984 samples and by ICP-OES in the 2012 samples.

### 2.4 Statistical analysis

Differences between the two sampling events 1984 and 2012 for soil chemistry (horizons: S 0–5, B 0–5, B 30–40 and B 80–90) and foliar chemistry were tested by paired sample *t*-tests. A repeated measures ANOVA was performed for each chemical soil parameter for each year separately (one way ANOVA, grouping factor: soil horizon) and results of multiple Bonferroni corrected paired comparison tests between the soil horizons were given. For the 1984 and 2012 data 4 horizons were compared with each other, while 6 soil horizons (S 0–5, S 10–20, B 0–5, B 10–20, B 30–40 and B 80–90) for the so-called 2012 extended data were used to evaluate whether heavy metals had moved down the soil profile. Furthermore, a one way ANOVA was performed to test differences between various forest site factors (geology, terrain, aspect) for each soil horizon and element separately and results of a Duncan multiple range test were given. Bivariate linear correlations were performed between total foliar nutrient contents and soil parameters for the years 1984 and 2012. All statistics were performed with the package IBM SPSS Statistics 21 software (IBM Corporation, Armonk, NY, US). Landscape maps of the geographical distribution of heavy metal contents in different soil layers and in the foliage were plotted with the Surfer 8 software (Golden Software LLC, Colorado, USA).

## 3 Results

### 3.1 Soil chemistry

**Fig. 2:**
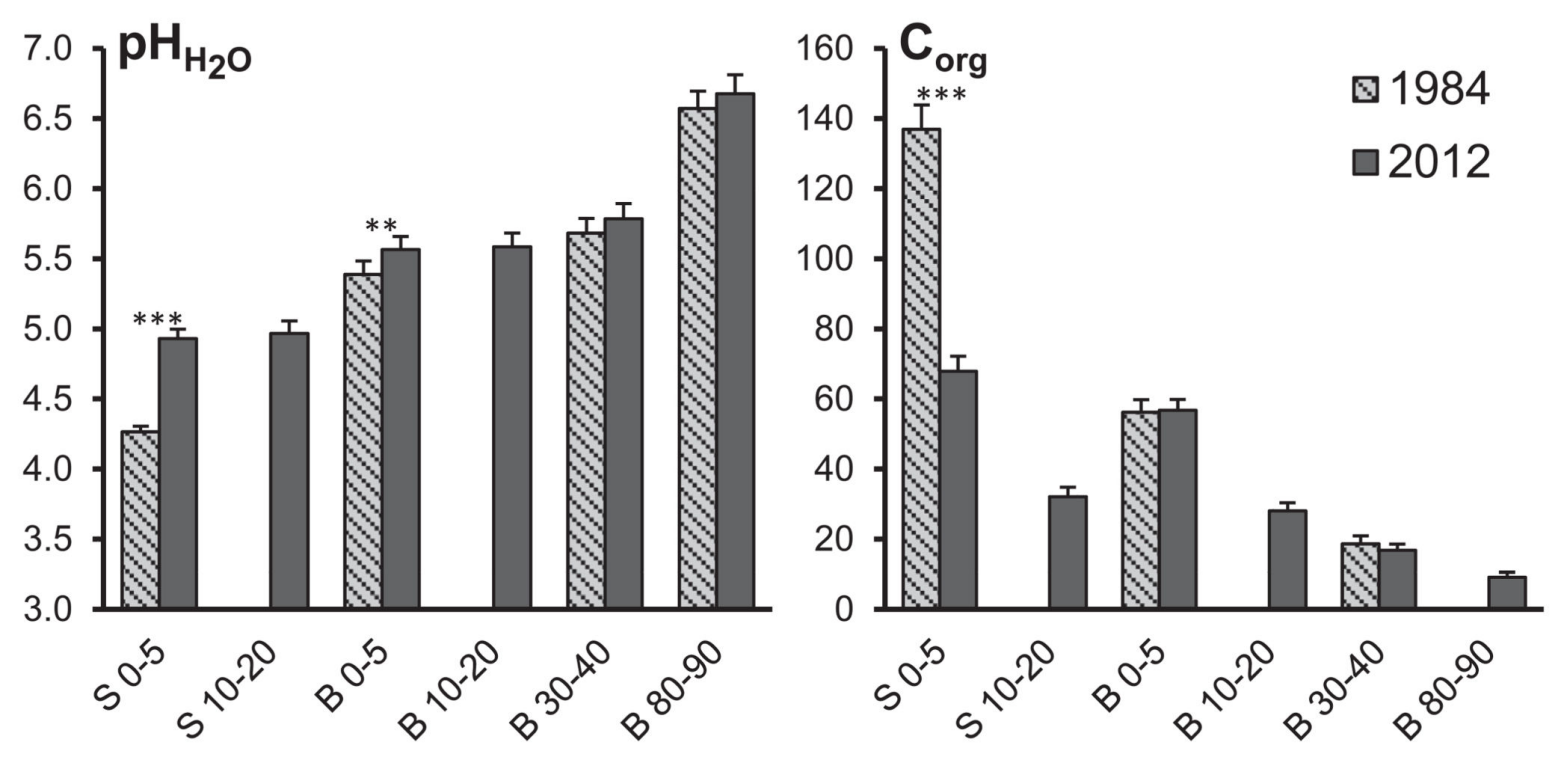
Mean soil pH (H_2_O) and C_org_ content (mg g^−1^) in different soil depths of the stemflow area (S 0–5 and S 10–20) and the between trees area (B 0–5, B 10–20, B 30–40 and B 80–90) at 97 beech stands in 1984 and 2012 (modified from Berger et al., 2016). Paired sample *t*-tests were performed to test significance of differences between the years 1984 and 2012 and only significant results are given as: **: p ≤ 0.01; ***: p ≤ 0.001. Error bars are ± S.E.

As pointed out above, we recently published part one of the overall study, focusing on soil acidification and macro nutrition of these beech stands (Berger et al., 2016). The most relevant previous results for understanding the biogeochemistry of heavy metals within the current second part are considered soil pH and C_org_ content, which were redrawn in Fig. 2. Soil pH (H_2_O) within the stemflow area (S 0–5) increased significantly by 0.6 units (4.3 to 4.9) from 1984 to 2012 (p < 0.001; as indicated by paired sample *t*-test). Soil pH (H_2_O) increased by 0.2 (5.4 to 5.6; p < 0.01) units at B 0–5 and there was a trend of slightly higher 2012 values in the deeper soil of the between trees area. Despite distinct signs of soil pH recovery in the stemflow area, present pH values were still lower than in the between trees area for comparable soil depths. Contents of C_org_ decreased markedly from 137 to 68 mg g^−1^ (p < 0.001) in the stemflow area (S 0–5) from 1984 to 2012, suggesting that this elemental loss was caused by improved mineralization rates of organic matter due to more favorable soil conditions (i.e, increased soil pH). On the other hand, a positive double-feedback between C_org_ and pH is likely, since mineralization of organic C and release of sequestered base cations will accelerate soil pH recovery in the top soil of the stemflow area. It must be pointed out that the present C_org_ content (57 mg g^−1^) at B 0–5 is still lower than at S 0–5.

Mean soil contents of Pb, Cu, Zn, Ni, Mn and Fe (mg kg^−1^) in the infiltration zone of stemflow near the base of the stem (S 0–5 and S 10–20) and in the between trees area (B 0–5, B 1020, B 30–40 and B 80–90; given ranges are soil depths in cm) at the 97 beech stands of the Vienna Woods in 1984 and 2012 are given in Table 1. Out of 24 cases (6 heavy metals x 4 soil depths) the 2012 values were smaller than the 1984 values in 19 cases (see paired *t*-tests statistics for significance in Table 1). Increasing metal contents over time were recorded in only 5 cases for Cu (B 0–5), Zn (S 0–5, B 0–5) and Mn (S 0–5, B 80–90). In general, declining atmospheric deposition of heavy metals over the past 3 decades was reflected in the soil, however, temporal changes of heavy metal contents were different for the individual elements.

**Fig. 3:**
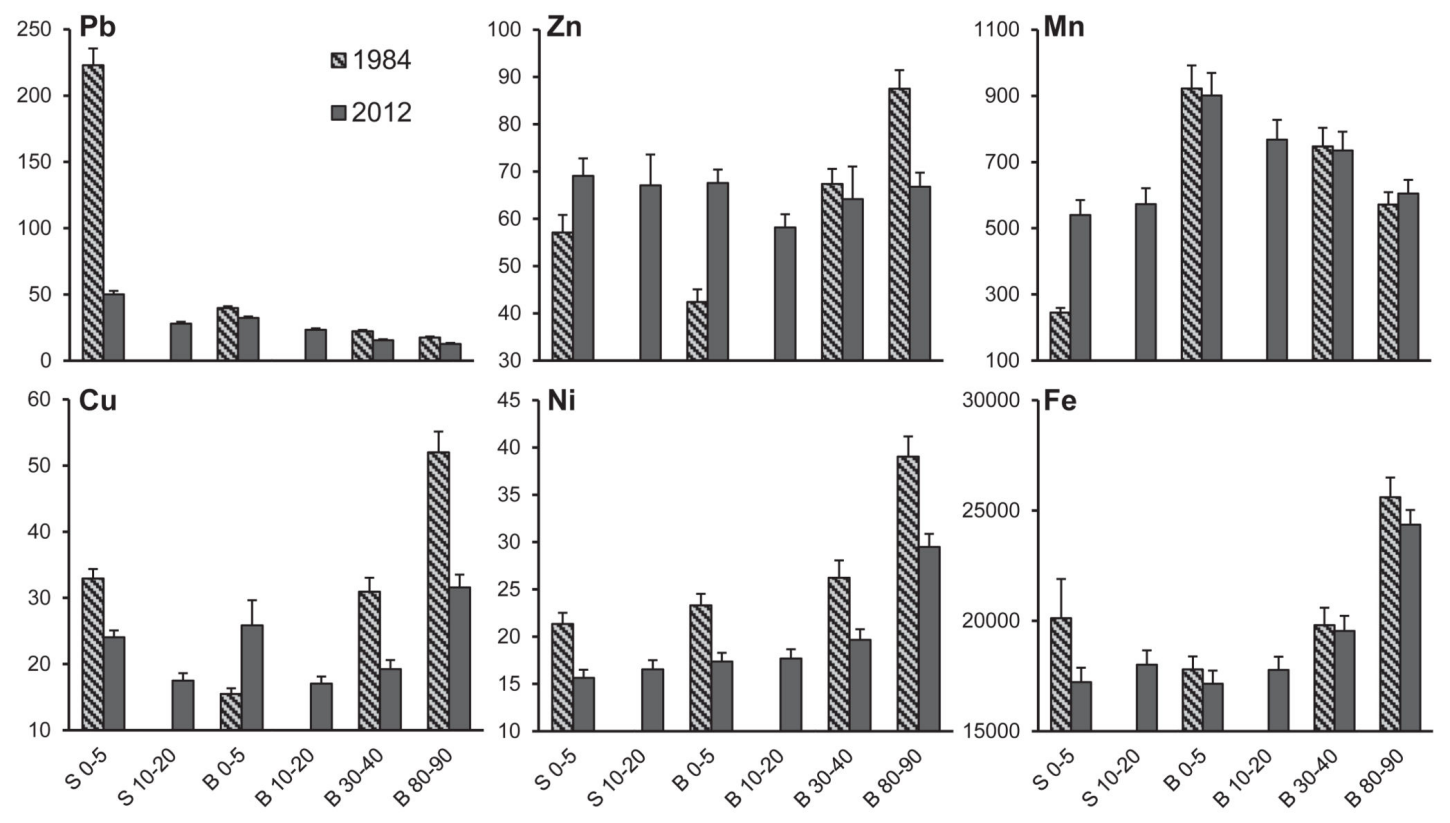
Mean soil contents of Pb, Cu, Zn, Ni, Mn and Fe (mg kg^−1^) in different soil depths of the stemflow area (S 0–5 and S 10–20) and the between trees area (B 0–5, B 10–20, B 30–40 and B 80–90) at 97 beech stands in 1984 and 2012. Error bars are ± S.E.

**Fig. 4:**
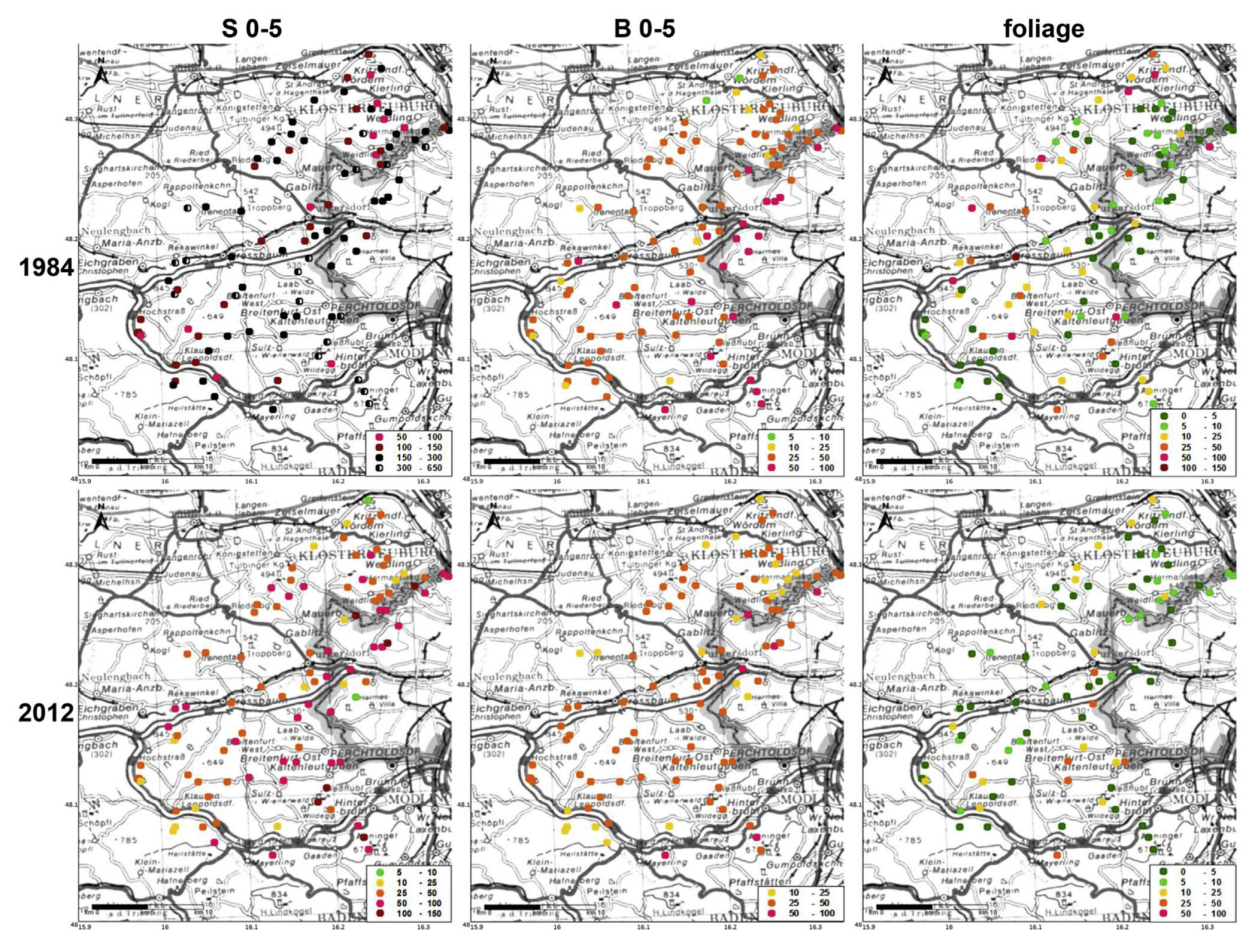
Geographical distribution of top soil Pb contents in the infiltration zone of stemflow (S 0–5; left) and the between trees area (B 0–5; middle), as well as foliar Pb contents (right), sorted by classes (mg kg^−1^), in beech stands of the Vienna Woods in 1984 (top) and 2012 (bottom).

The most striking result of this study was the decrease of Pb in the stemflow area from 223 to 50 mg kg^−1^ from 1984 to 2012, a decrease by 78% (p < 0.001; Table 1, Fig. 3). Similarly, at all soil depths in the between trees area, Pb contents were significantly lower in 2012 than in 1984. Despite distinct signs of an overall Pb decrease, present Pb contents in the stemflow area were still higher than in the between trees area for comparable soil depths (see different letters in Table 1). There is no doubt that this top soil enrichment of Pb in the stemflow area in 1984 was caused by atmospheric deposition from anthropogenic sources. In 2012, at nearly all beech stands measured soil Pb contents were lower than the proposed standard value for European norms (Rademacher, 2001; STable 1), except for the stemflow area at 6 sites (Fig. 4). Comparing the C_org_ pattern (Fig. 2) with the patterns of the 6 studied heavy metals shows the best match with Pb. This similarity is striking and supported by the highest positive bivariate Pearson correlation coefficients for the couple C_org_ and Pb in both years of the study (e.g.; S 0–5: 0.81 and 0.60; B 0–5: 0.53 and 0.44; for the years 1984 and 2012; p < 0.001 in all given cases).

**Fig. 5:**
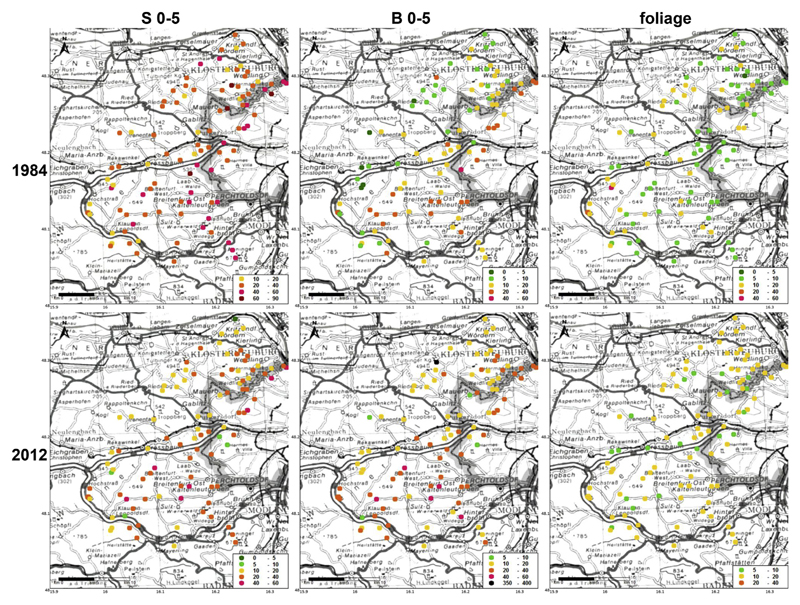
Geographical distribution of top soil Cu contents in the infiltration zone of stemflow (S 0–5; left) and the between trees area (B 0–5; middle), as well as foliar Cu contents (right), sorted by classes (mg kg^−1^), in beech stands of the Vienna Woods in 1984 (top) and 2012 (bottom).

Paired *t*-tests revealed significantly lower Cu contents in 2012 than in 1984 for S 0–5, B 30–40 and B 80–90. However, at B 0–5, Cu was significantly higher in 2012 than in 1984. The 2012 extended Cu values declined in the between trees area from 0–5 over 10–20 and increased thereafter over 30–40 to 80–90 cm soil depth (Table 1, Fig. 3). Such a pattern may indicate plant uptake of the micro nutrient in the densely rooted zone and top soil accumulation via litter and decomposition of plant biomass. In 2012, all soil data for Cu in the stemflow area (S 0–5) and at B 0–5 (with the exception of one extreme value which was kept in the data base; Fig. 5) were below the proposed standard value for European norms. At B 80–90, contents of Cu above 60 mg kg^−1^ were recorded at 9 beech stands (Table 2).

Total Zn contents showed a similar pattern as Cu in 1984: high contents in the stemflow area (S 0–5), low contents at B 0–5 and a steady increase from the top to the deep soil in the between trees area, suggesting geogenic origin (Table 1, Fig. 3). However, in 2012, contents of Zn were distributed evenly over all soil horizons, because top soil (S 0–5, B 0–5) contents increased and deep soil (B 80–90) contents decreased, suggesting plant cycling of the relatively mobile micro nutrient. The threshold value at which toxicity symptoms may occur (170 mg kg^−1^; Witter, 1992) was exceeded in 2012 at two stands (S 0–5) and one (B 0–5) stand, respectively (STable 1, SFig. 1).

Total Ni contents decreased markedly in the stemflow area (S 0–5) and the between trees area (B 0–5, B 30–40 and B 80–90) from 1984 to 2012 (Table 1). In both years, nickel was slightly lower in S 0–5 than in B 0–5 and increased significantly with soil depth within the between trees area, suggesting that geogenic origin plays a major role for this element as well. Nickel contents in the different soil layers were below the upper limit of the proposed standard value for European norms (60 mg kg^−1^) in all cases except for one site at B 80–90.

Although no significant changes were observed for Fe, total contents tended to decrease from 1984 to 2012 in the stemflow area (S 0–5) and the between trees area (B 0–5, B 30–40 and B 80–90). Contents of Fe increased with increasing soil depth in the between trees area (2012 extended data, Table 1, Fig. 3) and contents in the top soil layers (S 0–5, B 0–5) were similar in 2012.

Low Mn contents at S 0–5 in 1984 increased significantly until 2012 (Fig. 3). However, recent Mn contents were still higher at B 0–5 than at S 0–5 (as indicated by different letters in Table 1). The lower limit at which toxicity symptoms may occur (1500 mg kg^−1^; Kabata-Pendias and Pendias, 1984), was exceeded at 4 (S 0–5), 19 (B 0–5) and 10 (B 30–40) sites, respectively (STable 1).

### 3.2 Foliar chemistry

Mean foliar contents of Pb, Zn, Ni, Mn and Fe decreased significantly from 1984 to 2012 (Table 2). Contents of Cu tended to decrease, the element for which the emissions did hardly change over time (−1% from 1990 to 2012; EEA, 2014). Rademacher (2001) introduced a classification system for pollutants (Pb; 1: low, 2: adequate, 3: high) and micro nutrients (Cu, Zn, Mn, Fe, 1: deficient, 2: optimum, 3: surplus). The distribution of the studied beech stands within these three classes is summarized in Table 3. Though mean foliar Pb contents declined from 19 to 9 mg kg^−1^ (Table 2) over time, only one third of the studied beech stands were allocated in class 1 (low) in 2012. Critical Pb foliar levels (class 3) were still recorded for 24% of the sites. Recent foliar contents of Cu were within the surplus range (class 3), all other micro nutrients within the optimum range (class 2).

### 3.3 Relationship between soil and foliar chemistry

Bivariate Pearson correlation coefficients between foliar metal (Pb, Cu, Zn, Mn, Fe) content and the corresponding soil metal content at S 0–5 and B 0–5 for the years 1984 and 2012 were not significant in any of these 20 cases (5 metals x 2 horizons x 2 years). This finding supports the lack of a corresponding theory between soil chemical fertility (pollution) and plant response as found in part one of the overall study for macro nutrients (Berger et al., 2016). It must be pointed out, that in general, total soil cation contents are less useful than exchangeable cation soil contents for simulating plant availability. However, foliar Ni and soil Ni contents were significantly related with each other in 2012 (S 0–5: *R* = 0.35; p < 0.01; B 0–5: *R* = 0.21; p < 0.05; Ni was not measured in 1984).

### 3.4 Relationship between soil chemistry and site condition

Mean soil pH (H_2_O), Pb, Cu, Zn, Ni, Mn and Fe (mg kg^−1^) contents at S 0–5, B 0–5 and B 80–90 are given in Table 4 for the year 2012, grouped by geological parent material and terrain. Results of the one-way ANOVAs and posthoc Duncan multiple range test (different letters indicate significant differences, p < 0.05) indicate that soil pH and heavy metal contents differed significantly between the various types of the geological parent material. The only exception was found for Pb, which was not affected by the grouping variable geology in the deep soil (B 80–90). Soils on limestone (lime and dolomite) yielded the highest heavy metal contents in the stemflow area, indicating high accumulation rates due to low mobilization at high soil pH. In contrast, heavy metal soil contents on Flysch (Gaultflysch, Altlengbach beds, Greifenstein beds, Laab beds, Kahlenberg beds) were much lower. The highest contents of Cu, Zn and Mn were found on Flysch soils (B 80–90) developed on Greifensteiner-, Laab- and Kahlenberger beds.

The factor terrain was significant only for pH and Pb in the stemflow area (S 0–5). Soils on hilltops where characterized by the highest Pb soil contents and lowest soil pHs (Table 4). Since accumulation of Pb is usually increased at high soil pH, this fact strengthens that atmospheric Pb was preferentially deposited on wind exposed sites. Aspect affected soil contents in the stemflow area (S 0–5), which were enriched for Zn, Ni and Mn on northfacing- and to a lower extent on east- and southeast-facing slopes (data not shown). In all other cases, there was no impact of aspect on heavy metal soil contents in 2012.

### 3.5 Geographical distribution of soil and foliar heavy metal contents

Landscape maps of the geographical distribution of Pb, Cu and Zn in the top soil (S 0–5 and B 0–5) and in the foliage (sorted by element content classes) are plotted for the years 1984 and 2012 in Figs. 4 and 5 and SFig. 1, respectively.

In 1984, lead was preferentially accumulated in soils within the hilly terrain north and north-west of Vienna along the city boarder (Fig. 4). Higher Pb contents were also found in the south of the study area due to higher pH values in soils on limestone. The top soil showed the same pattern for S 0–5 and B 0–5, however, lead contents within the stemflow area were clearly enriched due to the filtering and funneling effect of the beech canopy. In 2012, the same distribution pattern was still recognizable, though at much lower soil contents. It is striking, that in 1984, foliage contents did not reflect the soil contents at all, there even seemed to be an opposite trend: the hilly terrain north and north-west of the City was characterized by decreased Pb foliar levels. This fact may be caused by high deposition of heavy metal elements onto the soil via fog (atmospheric inversion layer) during the leafless winter period at prevailing south to south-east winds from the City and by low deposition onto the leaves during the summer at prevailing west winds. Foliar contents of Pb were slightly increased in the valleys along the main traffic roads.

Spatial distribution patterns of Cu soil contents (S 0–5, B 0–5) did not change between 1984 and 2012, though Cu soil contents were absolutely higher in 1984 (Fig. 5). Patterns of Cu were similar to Pb, indicating accumulation in the hilly terrain north and northwest of Vienna along the city boarder and in the south on limestone. Foliar Cu contents did not show any clear spatial patterns, probably because most values in 2012 were within a small range (6.5–14.9 mg kg^−1^; Table 2). The fact that most sites changed from the class 5–10 to 10–20 mg kg^−1^ over time without a significant change of the overall mean between the two years (Table 2) was caused by a few extreme values in 1984 (see above).

Again, a similar pattern was recorded for soil contents of Zn in the top soil in 1984 with clear signs of accumulation around the city boarder (SFig. 1). While differences between S 0–5 and B 0–5 were clear in 1984 (compare Fig. 3), these differences ceased in 2012 and Zn soil contents were spread more evenly throughout the study area. Though soil Zn contents were not correlated with foliar Zn contents (see above) in 1984, high foliar Zn levels were concentrated around the northern city boarder as well and along the highways A1 and A21. No clear spatial patterns of foliar Zn contents were visible in 2012.

## 4 Discussion

### 4.1 Have soil contents of Pb, Zn, Cu, Ni, Mn and Fe changed as atmospheric heavy metal deposition declined over the last three decades?

By phasing out leaded petrol in Austria between the mid-1980s and 1993 a general decline of emissions and subsequent deposition of Pb resulted in a decrease of Pb contents in soils. Lead contents in forest soils decreased since the implementation of environmental protection measures to improve the air quality in the United States and Europe (Block and Gauer, 2012; Evans et al., 2005; Gubler et al., 2015; Johnson and Richter, 2010; Schubert et al., 2015; Smidt et al., 2012). The most striking result of this study was the decrease of Pb in the stemflow area from 223 to 50 mg kg^−1^ from 1984 to 2012, a decrease by 78% (Fig. 3), which was basically expected in the light of reported temporal trends in vehicular and industrial emissions in Austria and cited results of other studies that compared present and past heavy metal contents in soils. In 1984, the threshold value, at which toxicity symptoms may occur (>100 mg kg^−1^), was exceeded at 86 out of 97 top soil samples within the infiltration zone of stemflow (S 0–5). However, in 2012, only 6 beech stands exceeded this value (STable 1). Mean Pb contents of the top soil within the between trees area (B 0–5) decreased from 40 (1984) to 32 (2012) mg kg^−1^. Lead contents in the top soils of the Vienna Woods were similar or slightly lower in comparison with other areas in Europe, amounting 41–44 and 30 mg kg^−1^ in forest top soils of western and southern Germany and Switzerland, respectively (Block and Gauer, 2012; Gubler et al., 2015; Schubert et al., 2015). Results from the Austrian Forest Soil Monitoring System showed a very distinct decrease of Pb in the forest floor and to a lower extent in the top mineral soil between the first (1987/89) and the second (2006/07) soil inventory (Mutsch, 1992, 1998; Smidt et al., 2012). It must be pointed out that in none of these cited European studies heavy metal soil chemistry of identical monospecific forest stands, characterized by uniform site conditions, was monitored over such a long time span without any methodologically changes as was done in the present research.

Comparing two sampling campaigns over such a long time span has to be tested critically, since the beech stands grew older which might have affected the heavy metal content in the stemflow area by increasing stemflow volume and, secondly, thinning might have affected the between trees area as well. However, the periodic annual increment in volume of a forest stand increases to a maximum value as a tree matures and then declines during the rest of the silvicultural cycle. Since the selected beech stands were older than 80 years in 1984, the stands had passed the period of maximum growth already before the beginning of the study period (Jonard et al., 2015). Because beech is known to achieve full canopy closure at low stand density, the last thinning is usually done before the age of 80 as so-called “light growth thinning”. In fact, our study stands were not thinned between 1984 and 2012, since no stumps were visible. According to Berger et al. (2008), beech stemflow is a function of the crown projection area of the individual beech tree. Hence, it is justified to assume that neither the stemflow area was affected by increased stemflow volume nor the between trees area by thinning between 1984 and 2012. Finally, we tested possible stand age effects: results of an ANOVA revealed no significant differences of Pb contents for S 0–5 or B 0–5 between the age classes (in the year 1984) 80–90, 90–100, 100–110, 110–120, 120–130, 130–140, 140–150, 150–160, supporting the above assumptions.

In both sampling years, the highest Pb contents were found in the top soil samples (S 0–5 and B 0–5) and Pb contents decreased significantly with depth in the between trees area (Table 1). Topsoil enrichment at forested sites points to an anthropogenic origin of Pb via long range transport of pollutants. This finding is consistent with other studies deducing anthropogenic sources of Pb from vertical distribution pattern in soils (Clemente et al., 2008; Fiala et al., 2008; Hernandez et al., 2003; Luster et al., 2006; Neupane and Roberts, 2009).

Berger and Muras (2016) hypothesized that the micro-spatial heterogeneity of soil columns downhill of a beech stem is a function of historic acid loads (stem area received much higher deposition loads in the past than the between trees area) and time (a space-for-time substitution due to higher soil solution fluxes close to the stem). This hypothesis was strengthened by results of part one of the overall study (e.g., compare quick pH recovery at S 0–5, Fig. 2, Berger et al., 2016) and seems to concur well with our data, since Pb contents decreased substantially in the infiltration zone of stemflow compared to the between trees area. However, despite distinct signs of soil recovery and declining heavy metal contents, present Pb contents in the stemflow area were still higher than in the between trees area after 3 decades of decreased Pb deposition (Table 1, Fig. 2). Similar results, when comparing top soil samples at different distances from the trunk, were found by Bini and Bresolin (1998), Józwiak et al. (2013) and Wilcke et al. (1998). Accumulation of trace metals in the stemflow area was reported in the 1980s for soils in the Vienna Woods by Glatzel and Kazda (1985) and Kazda et al. (1986) as well.

The observed decrease of Pb contents at S 0–5 was striking, raising the question where this metal has gone. Enhanced soil acidification may also affect the sub-surface horizons by a further downward movement of metals previously released from the humus layer (Steinnes and Friedland, 2006). We do not have any information about forest floor Pb contents but these amounts are expected small at mull sites. E.g., forest floor C_org_ stocks at 3 beech sites on Flysch within the study area amounted 13–16% of the C_org_ stocks sequestered in 0–10 cm soil depth (Muras, 2012; note that C_org_ and Pb contents were highly correlated with each other as stated above). Within the stemflow area, we sampled only till a soil depth of 10–20 cm, since many authors stated that movement of Pb out of the mineral soil was rather slow and a significant loss of Pb from the top mineral soil would take a century or even more (e.g., Johnson et al., 1995; Steinnes and Friedland, 2006). Assuming the same bulk densities in 0–5 and 10–20 cm, what is justified for these loamy to clayey soils, the lost amounts of Pb (deduced from Pb contents in Fig. 3) were not transported down to 20 cm soils depth and a further transport seems unlikely. Therefore, we suggest that small erosion processes due to high stemflow fluxes caused this decline in the stemflow area rather than a movement of Pb down the soil profile. Probably, most of the lost C_org_ (see Fig. 2) was mineralized and emitted via soil CO_2_ efflux. Hence, we hypothesize that the remaining Pb, which used to be highly linked with C_org_, became subject of additionally increased erosion by surface runoff and dilution effects after associated loss of soil aggregate stability.

Between 1990 and 2012, emissions of Cu, Zn and Ni decreased by 1%, 42% and 67%, respectively (EEA, 2014). This general decline resulted in a decrease of these metals in the studied soil horizons between 1984 and 2012, except for Cu at B 0–5 and Zn at S 0–5 and B 0–5 (Table 1, Fig. 3). Mosses, known to be useful bioindicators for air quality, revealed significant decreases of these elements for an Austrian wide grid from 1991 to 2005 as well (Cu: −7.1%; Zn: −21.6%; Ni: −51.4%). However, north-eastern parts of the country were still highly effected by cross-border pollution and increasing traffic emissions of Austria's capital city Vienna (Zechmeister et al., 2008). Our recent soil survey did not show any significant contamination for theses metals, since all data were below the proposed standard value for European norms (Rademacher, 2001) and far below toxic values (Kabata-Pendias and Pendias, 1984).

Top soil accumulation of Cu and Zn via litter and decomposition of plant biomass, which was enriched by these elements during the last decades, and losses in deeper soil horizons within the between trees area between 1984 and 2012 indicate plant uptake. Increased mobility during the Acid Rain period at lower soil pH (Clemente et al., 2008; Scheffer and Schachtschabel, 2010) caused increasing element contents with increasing soil depth in 1984. Constant return of these metals via nutrient cycling and increasing soil pH lead to an effective reduction of mobility for these elements (Luster et al., 2006). Consequently, contents of Cu and Zn were distributed more evenly over all soil horizons in 2012 (Fig. 3).

In both sampling years, Ni contents increased from the top soil to the deep soil in the between trees area. This fact may indicate leaching within the soil profile. According to Luster et al. (2006) mobility of Ni is high in the pH range 4–5. In both years, nickel was slightly lower in S 0–5 than in B 0–5 suggesting that Ni via atmospheric deposition was less important than Ni of geogenic origin.

Mean Mn and Fe contents in the studied forest soils of the Vienna Woods did not change significantly over time (Table 1, Fig. 3), except Mn contents increased at S 0–5 from 1984 to 2012. Because Mn and Fe are the most abundant elements in the lithosphere (Alloway, 2013; Kabata-Pendias and Pendias, 1984) and atmospheric deposition of these elements is low, the role of Mn and Fe as indicator for soil pollution is considered less important. The lower Mn contents at S 0–5 in 1984 were caused by soil leaching at low soil pH, as reported by Blake and Goulding (2002) and Skrivan et al. (1995). Meanwhile (2012), Mn soil contents at S 0–5 increased via plant cycling at improved soil conditions (Fig. 3).

Finally, we can answer our research question 1, that declining atmospheric deposition of heavy metals were reflected in the soil. The exclusive increases over time in the top soil for Zn and Mn in the stemflow area and for Cu and Zn in the between trees area were caused by plant nutrient cycling at improved soil conditions. Long-term changes of heavy metal distribution were different for the individual elements.

### 4.2 Are heavy metal soil contents related to forest site factors (geography, geology, terrain, aspect)?

The study sites were selected in a forested landscape, characterized by quite different atmospheric pollution loads, since their location ranges from the boarder of a large metropolitan area in the central east (Vienna) to remote regions in the western parts of the Vienna Woods. Sources of heavy metal deposition from urban environments are mainly traffic, industry and burning of fossil fuels and municipal wastes. Traffic causes the major sources of Pb (burning of leaded fuel until the introduction of unleaded gasoline), Cu (abrasion of brakes) and Zn (abrasion of tires; Biasioli et al., 2006; Mielke et al., 1999; Van Bohemen and Janssen Van de Laak, 2003).

The impact of the city Vienna on heavy metal soil contents could be nicely mapped for Pb (Fig. 4), Cu (Fig. 5) and Zn (SFig. 1). Top soil accumulation of these metals around the city boarder was very clear in 1984 and attenuated till 2012 due to declining emission rates. The highest metal soil contents were recorded within the hilly terrain north and north-west of Vienna caused by occult deposition via fog (atmospheric inversion layer) during fall and winter at prevailing south to south-east winds. A second region, characterized by high heavy metal soil contents, has been the southern part of the Vienna Woods on limestone. However, this result was less caused by high deposition rates but rather by high accumulation rates due to low mobility and losses over time at high soil pH. For example, the mean soil Pb ratio (S 0–5/B 0–5; i.e.: soil Pb content in S 0–5, divided by that in B 0–5) was significantly higher on limestone (2.18; lime and dolomite) than the corresponding ratios on the 5 geological parent materials on Flysch (1.39–1.65; no significant differences; Table 4) in 2012. The fact that soil pH (B 0–5) was positively correlated (p < 0.01; *N* = 97) with soil Pb ratio (S 0–5/B 0–5) in 2012, justifies to ascribe lower mobility to higher soil pH.

Heavy metal soil contents and soil pH differed significantly between the various types of the geological parent material (Table 4). The only exception was found for Pb, which was not affected by the grouping variable geology in the deep soil (B 80–90). This finding supports the theory that the observed top soil distribution patterns of Pb were caused by atmospheric deposition and not by geogenic origin. In all other cases, it is difficult to disentangle these two different types of sources. In general, this problem can be solved by looking at the elemental soil differences stemflow area minus between trees area and top soil minus deep soil, which turned out to be good indicators for atmospheric pollution. On the other hand, higher contents of some heavy metals at greater depth are likely not to represent geogenic sources but might be ascribed to higher soil pH values and lower C_org_ contents at greater depth which would reduce heavy metal mobility and thus lead to a long-term enrichment compared to the topsoil.

In 1984, soils on hilltops and upper slopes showed significantly higher Pb-, Cu- and Zn soil contents at S 0–5 for hilltops and upper slopes than for middle and lower slopes (not shown). Three decades (2012) later the factor terrain was significant only for Pb in the stemflow area (S 0–5; Table 4). Since soils on hilltops where characterized by the lowest soil pHs and accumulation of heavy metals is increased at high soil pH, this fact strengthens that atmospheric Pb was preferentially deposited on wind exposed sites. However, the impact of aspect on heavy metal soil contents was not significant except for Zn, Ni and Mn at S 0–5.

Finally, we can answer our research question 2 that heavy metal soil contents were related to the forest site factors geography, geology, terrain and aspect. However, since these factors are interrelated to each other, it is a challenge to separate anthropogenic-from geogenic heavy metal sources.

### 4.3 Are beech (Fagus sylvatica) foliar contents useful as bioindicators for heavy metal contamination of soils?

Reduction of emissions of heavy metals and of acidifying compounds (that promoted soil mobility of heavy metals) at an international level were expected to decrease uptake rates of heavy metals by plants. In fact, mean foliar contents of Pb, Zn, Ni, Mn and Fe decreased significantly from 1984 to 2012 (Table 2) and contents of Cu tended to decrease. Decreasing foliar Pb contents over time were observed in Europe, Italy and SW Germany as well (Fantozzi et al., 2013; Franzaring et al., 2010; Rademacher, 2001). Declining heavy metal contents in mosses across Europe were reported by Harmens et al. (2015). Lead is the only non-essential, potentially toxic trace element of the studied metals. Critical Pb foliar levels were still recorded for 24% of the sites. Recent foliar contents of Cu were within the surplus range, all other micro nutrients within the optimum range (Table 3). Relatively mobile elements like Zn or Mn can be taken up by the root system and transported to the canopy, while high foliage contents of less mobile elements like Pb are usually the result of direct deposition on the leaf surface and consequently good indicators of regional pollution levels (Rademacher, 2001).

Pearson correlation coefficients between foliar metal (Pb, Cu, Zn, Mn, Fe) content and the corresponding soil metal content were not significant. This finding clearly indicates a lack of a corresponding theory between soil chemical fertility (pollution) and plant response as found in part one of the overall study for macro nutrients (Berger et al., 2016). Hence, in our study heavy metal foliar contents were not useful as bioindicators for contaminated soils. As pointed out above, heavy metal foliar contents may be useful as bioindicators for air pollution of elements that are not taken up via the root system but stick to the leaf surface via dry deposition processes. Theoretically, this should be the case for Pb, but in our case dry deposition sources were quite different between the vegetation- and the leafless period, causing quite different patterns between soil and foliage contents as well.

Finally, we can simply answer our research question 3 that heavy metal beech foliar contents were not useful as bioindicators for heavy metal contamination of soils. However, heavy metal foliar contents may be useful as bioindicators for atmospheric pollutants that accumulate in the foliage not via the soil- but via the air path.

## 5 Conclusions

We resampled 97 old-growth beech stands in the Vienna Woods in Austria. This study exploits an extensive data set of soil (infiltration zone of stemflow and between trees area) and foliar chemistry of the heavy metals Pb, Cu, Zn, Ni, Mn and Fe from 1984 to 2012. International implementation of emission controls caused declining deposition of heavy metals which was reflected in the studied soils. The exclusive increases over time in the top soil for Zn and Mn in the stemflow area and for Cu and Zn in the between trees area are assumed to be caused by plant nutrient cycling at improved soil conditions due to recovery from Acid Rain. Long-term changes of heavy metal distribution were different for the individual elements. Heavy metal soil contents were related to the forest site factors geography, geology, terrain and aspect. Because these site factors are interrelated to each other, it is a challenge to separate anthropogenic-from geogenic heavy metal sources. Elemental soil differences stemflow area minus between trees area and top soil minus deep soil turned out to be good indicators for atmospheric pollution. Heavy metal foliar contents did not correlate with the corresponding element in the soil but may be useful as bioindicators for atmospheric pollutants that accumulate in the foliage not via the soil- but via the air path. Mean foliar contents of all six studied heavy metals decreased within the last three decades, but plant supply with the micro nutrients Cu, Zn, Mn and Fe is still in the optimum range for beech trees. It is concluded that heavy metal pollution is not critical for the studied beech stands any longer. But, microsites, affected by beech stemflow, indicate critical values up to present and are very useful for studying the legacy of high atmospheric heavy metal deposition.

## Appendix A. Supplementary data

Supplementary data related to this article can be found at http://dx.doi.org/10.1016/j.envpol.2017.06.080.

Supplemental data

## Figures and Tables

**Table 1:** Mean total contents of Pb, Cu, Zn, Ni, Mn and Fe (mg kg^−1^) in the infiltration zone of stemflow near the base of the stem (S 0–5 and S 10–20) and in the between trees area (B 0–5, B 10–20, B 30–40 and B 80–90; given ranges are soil depths in cm) at 97 beech stands of the Vienna Woods in 1984 and 2012.

Element	S 0–5		S 10–20	B 0–5		B 10–20	B 30–40		B 80–90	
				
	1984	2012	2012	1984	2012	2012	1984	2012	1984	2012
*Pb*	222.9	50.0	28.0	39.7	32.3	23.4	22.2	15.5	17.4	12.6
paired *t*-test	***			***			***		***	
multiple comparison 1984	D			C			B		A	
multiple comparison 2012		d			c			b		a
multiple comparison 2012 extended		*f*	*d*		*e*	*c*		*b*		*a*
*Cu*	32.9	24.0	17.5	15.5	25.8	17.0	30.9	19.3	52.0	31.6
paired *t*-test	***			**			***		***	
multiple comparison 1984	B			A			B		C	
multiple comparison 2012		b			abc			a		c
multiple comparison 2012 extended		*c*	*a*		*abcd*	*a*		*b*		*d*
*Zn*	57.1	69.1	67.1	42.4	67.6	58.2	67.4	64.2	87.5	66.8
paired *t*-test	**			***			n.s.		***	
multiple comparison 1984	B			A			C		D	
multiple comparison 2012		a			a			a		a
multiple comparison 2012 extended		*a*	*a*		*a*	*a*		*a*		*a*
*Ni*	21.3	15.6	16.5	23.3	17.4	17.7	26.2	19.7	39.0	29.5
paired *t*-test	***			***			***		***	
multiple comparison 1984	A			AB			B		C	
multiple comparison 2012		a			b			c		d
multiple comparison 2012 extended		*a*	*ab*		*b*	*b*		*c*		*d*
*Mn*	245.2	540.2	572.5	922.3	901.3	767.6	747.1	735.1	571.5	604.9
paired *t*-test	***			n.s.			n.s.		n.s.	
multiple comparison 1984	A			D			C		B	
multiple comparison 2012		a			c			b		ab
multiple comparison 2012 extended		*a*	*a*		*d*	*cd*		*bc*		*ab*
*Fe*	20120.1	17225.0	18016.9	17799.8	17150.5	17778.3	19804.0	19547.6	25606.6	24363.6
paired *t*-test	n.s.			n.s.			n.s.		n.s.	
multiple comparison 1984	AB			A			B		C	
multiple comparison 2012		a			a			b		c
multiple comparison 2012 extended		*a*	*bc*		*ab*	*ac*		*d*		*e*

Paired sample *t*-tests were performed to test significance of differences between the years 1984 and 2012: ns: not significant; *: p ≤ 0.05; **: p ≤ 0.01; ***: p ≤ 0.001. A repeated measures ANOVA was performed for each parameter and year separately and results of multiple Bonferroni corrected paired comparison tests between the soil horizons are given (different letters indicate significant differences, p < 0.05; A, a and *a* represents the lowest mean of 1984, 2012 and 2012 extended, respectively).

**Table 2:** Mean element contents, standard errors and ranges (mg kg^−1^) of fresh beech foliage in 1984 and 2012.

Element	Year	Mean	SE	Range	paired *t*-test
Pb	1984	19.2	2.6	1.7–128.6	**
	2012	9.2	1.2	0.1–44.0	
Cu	1984	11.5	0.9	1.1–72.6	n.s.
	2012	11.0	0.2	6.5–14.9	
Zn	1984	50.0	3.2	22.8–305.9	***
	2012	35.2	1.5	15.3–91.8	
Ni	1984				
	2012	7.4	0.2	4.0–12.0	
Mn	1984	1051.1	71.6	29.4–4591.1	***
	2012	828.0	61.7	16.4–3352.9	
Fe	1984	205.0	7.3	112.6–468.1	***
	2012	115.8	2.7	51.2–177.2	

Paired sample *t*-tests were performed to test significance of differences between the years 1984 and 2012: ns: not significant; *: p ≤ 0.05; **: p ≤ 0.01; ***: p ≤ 0.001.

**Table 3:** Percentage of sites (*N* = 97) in 2012, classified by foliar contents of Pb, Cu, Zn, Mn and Fe (mg kg^−1^) in (1) low/deficient-, (2) adequate/optimum- and (3) high/surplus range (pollutant: Pb/micro nutrient: Cu, Zn, Mn, Fe) according to Rademacher (2001).

Year	Pb			Cu			Zn			Mn			Fe		
Class	1	2	3	1	2	3	1	2	3	1	2	3	1	2	3
Limit	≤2	2–10	>10	≤5	5–10	>10	≤20	20–50	>50	≤60	60–2500	>2500	≤60	60–200	>200
1984	6	54	40	1	60	39	0	69	31	0	96	4	0	57	43
2012	33	43	24	0	20	80	5	81	14	6	92	2	6	94	0

**Table 4:** Mean soil pH (H_2_O), Pb, Cu, Zn, Ni, Mn and Fe (mg kg^−1^) contents in different soil horizons (stemflow area: S 0–5; between trees area: B 0–5, B 80–90) grouped by geological parent material and terrain in beech stands of the Vienna Woods in 2012.

Soil horizon	pH		Pb		Cu		Zn		Ni		Mn		Fe	
*S 0-5*	*Geology*													
Gaultflysch (*N* = 15)	4.4	a	42.1	a	16.2	a	37.9	a	8.0	a	282.9	a	11442.7	a
Altlengbach beds (*N* = 6)	4.6	a	34.9	a	15.3	a	43.9	a	8.1	a	272.5	a	12236.9	a
Greifenstein beds (*N* = 5)	4.9	a	51.0	a	22.4	ab	48.3	ab	13.0	b	410.8	ab	15159.9	ab
Laab beds (*N* = 32)	4.9	a	42.9	a	27.1	b	69.9	b	18.8	bc	734.9	b	19176.2	b
Kahlenberg beds (*N* = 31)	4.9	a	54.4	a	25.8	b	73.4	b	15.5	b	509.9	ab	17923.4	b
Lime and dolomite (*N* = 8)	6.3	b	87.2	b	27.5	b	139.5	c	25.5	c	642.1	b	22588.2	b
*B 0-5*														
Gaultflysch	4.7	a	29.7	ab	14.9	a	50.7	a	8.1	a	531.2	a	10949.2	a
Altlengbach beds	4.9	ab	24.9	a	14.5	a	42.8	a	9.8	a	471.8	a	12740.5	ab
Greifenstein beds	5.5	bc	34.4	b	89.6	c	79.7	b	15.3	b	750.7	ab	15602.8	bc
Laab beds	5.5	bc	32.3	ab	28.0	bc	68.3	b	21.4	b	1176.8	b	20020.3	c
Kahlenberg beds	5.8	c	32.4	ab	22.1	ab	74.7	b	17.9	b	969.5	ab	17734.2	c
Lime and dolomite	7.3	d	41.5	b	21.0	ab	79.8	b	23.9	b	679.5	ab	19670.0	c
*B 80-90*														
Gaultflysch	5.4	a	10.3	a	21.4	ab	55.2	b	19.4	a	362.5	ab	22183.6	b
Altlengbach beds	5.7	a	8.9	a	21.3	ab	55.8	bc	21.7	ab	220.7	a	23986.2	b
Greifenstein beds	6.8	bc	12.8	a	40.8	c	60.0	bc	34.7	b	553.6	c	24633.9	b
Laab beds	6.2	ab	15.2	a	42.2	c	76.3	c	36.0	b	716.6	c	26715.9	b
Kahlenberg beds	7.6	cd	11.3	a	30.2	bc	74.3	bc	31.3	b	735.0	c	25642.0	b
Lime and dolomite	8.3	d	13.6	a	13.4	a	36.6	a	17.7	a	479.3	bc	14729.1	a
*S 0-5*	*Terrain*													
Lower slope (*N* = 13)	4.8	ab	43.3	a	22.3	a	58.7	a	14.6	a	419.5	a	18197.4	a
Middle slope (*N* = 43)	5.0	b	45.1	a	24.4	a	68.9	a	15.6	a	555.8	a	16838.9	a
Upper slope (*N* = 31)	5.0	ab	54.6	ab	23.4	a	72.5	a	16.6	a	555.5	a	17492.4	a
Hilltop (*N* = 10)	4.5	a	72.3	b	29.3	a	77.9	a	15.3	a	616.3	a	17472.9	a
*B 0-5*														
Lower slope	5.7	a	29.0	a	19.2	a	66.1	a	16.5	a	910.9	a	17588.8	a
Middle slope	5.6	a	31.4	a	31.6	a	68.3	a	17.8	a	842.2	a	17293.5	a
Upper slope	5.6	a	34.1	a	21.3	a	64.6	a	17.7	a	904.3	a	17159.9	a
Hilltop	5.2	a	36.6	a	24.1	a	78.4	a	16.0	a	1233.5	a	15963.4	a

A one-way ANOVA (geology and terrain, respectively) was performed for each soil horizon and element separately and results of a Duncan multiple range test are given (different letters indicate significant differences, p < 0.05; a represents the lowest mean).
