# Supplementary material for: Declining atmospheric deposition of heavy metals over the last three decades is reflected in soil and foliage of 97 beech (Fagus sylvatica) stands in the Vienna Woods
Source: Environ Pollut. Author manuscript; Available in PMC 2017 Nov 1. (PMC5584674; doi:10.1016/j.envpol.2017.06.080)
Supplement: Supplemental data [file NIHMS73543-supplement-Supplemental_data.pdf]

Supplementary Material

Declining atmospheric deposition of heavy metals over the last three decades is reflected in soil and foliage of 97 beech (*Fagus sylvatica*) stands in the Vienna Woods

Selina Türtscher, Pétra Berger, Leopold Lindebner and Torsten W. Berger
Department of Forest- and Soil Sciences, Institute of Forest Ecology, University of Natural Resources and Live Sciences (BOKU),
Peter Jordan-Straße 82, 1190 Vienna, Austria

STable 1 Ranges of total soil contents of Pb, Cu, Zn, Ni, Mn and Fe in the infiltration zone of stemflow (S 0-5) and the between trees area (B 0-5 and B 80-90) at 97 beech stands of the Vienna Woods in 2012 and standard values of heavy metals for soils (mg kg^{-1}).

Element	S 0-5	B 0-5	B 80-90	Standard value[1)]	Background in soils[2)]	Earth´s crust[3)]	Toxic value[4)]
Pb	7.9 - 146.4	12.1 - 66.2	0.2 - 51.6	50 - 100	17	14	100 - 400
Cu	4.2 - 52.5	9.1 - 375.7	2.3 - 91.9	30 - 60	2 - 50	50	60
Zn	18.4 - 188.6	23.5 - 181.7	16.4 - 166.1	100 - 200	10 - 100	75	170
Ni	3.2 - 36.9	2.8 - 44.5	4.0 - 60.7	30 - 60	3 - 48	80	95
Mn	31.2 - 1853.9	20.4 - 2749.3	114.4 - 1777.2		850	950	1500 - 3000
Fe	2524.9 - 31535.1	3286.1 - 28996.1	6941.2 - 39720.6				

[1)] European norms (Rademacher, 2001).
[2)] Global average background concentration in soil (Alloway, 2013).
[3)] Sparks (2003).
[4)] Threshold value at which toxicity symptoms may occur (Van Mechelen et al., 1997; Witter, 1992; Kabata-Pendias and Pendias, 1984).

Alloway, B.J., 2013. Heavy metals in soils, third ed. Springer, Netherlands, p. 614. ISBN 978-94-007-4469-1.
Kabata-Pendia, A., Pendias, H., 1984. Trace Elements in Soils and Plants. CRC Press, Boca Raton.
Rademacher, P., 2001. Atmospheric Heavy Metals and Forest Ecosystems. UN/ECE, Geneva, p. 75.
Sparks, D.L., 2003. Environmental Soil Chemistry, second ed. Academic Press, USA, p. 367.
Van Mechelen, L., Groenemans, R., van Ranst, E., 1997. Forest soil condition in Europe: results of a large soil survey. Technical Report. EC-UN/ECE, Brussels Geneva, p. 279.
Witter, E., 1992. Heavy metal concentrations in agricultural soils critical to microorganisms. Report 4079. Swedish Environmental Protection Agency, Sweden, p. 44.

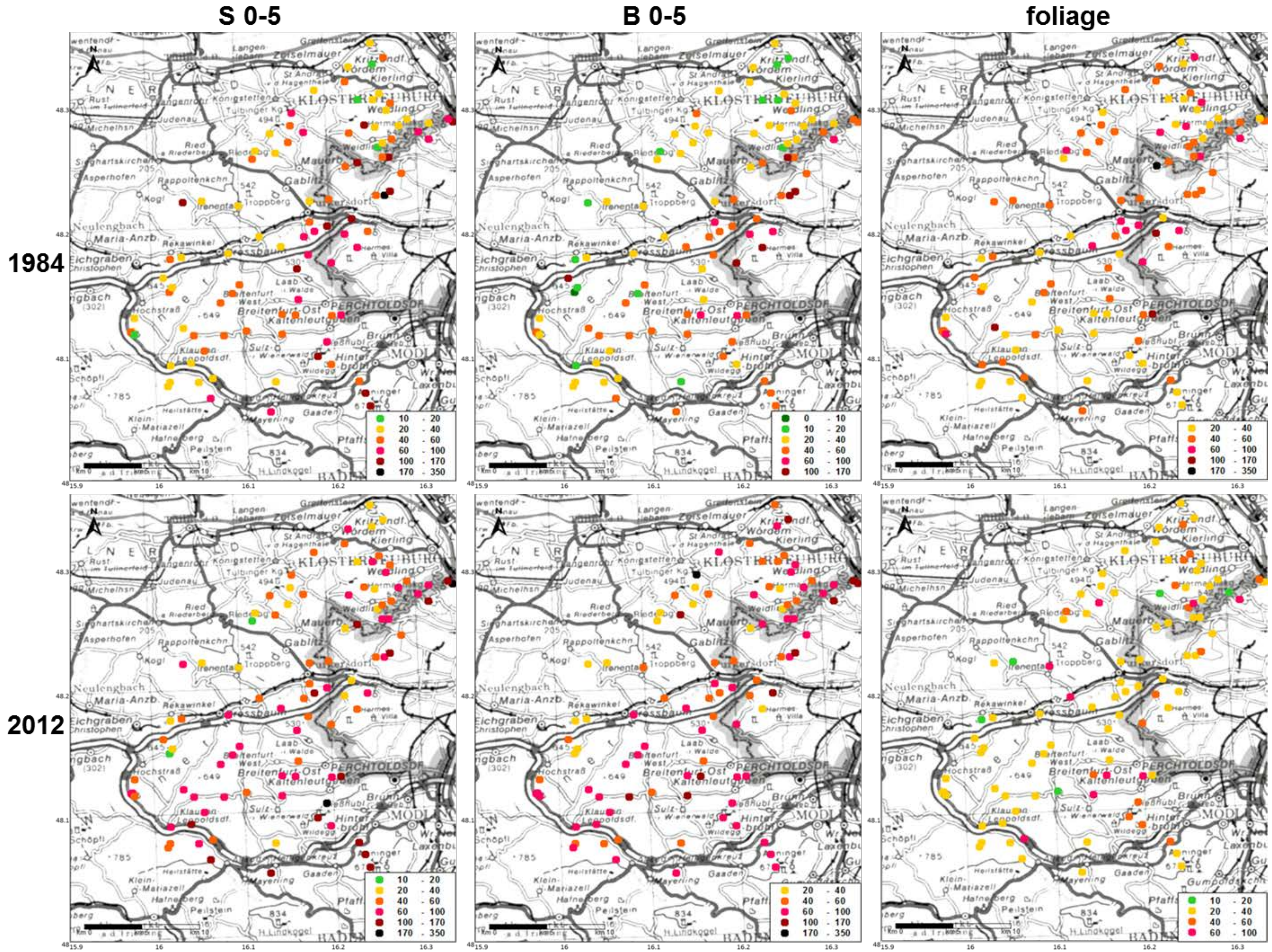

SFig. 1. Geographical distribution of top soil Zn contents in the infiltration zone of stemflow (S 0-5; left) and the between trees area (B 0-5; middle), as well as foliar Zn contents (right), sorted by classes (mg kg^{-1}), in beech stands of the Vienna Woods in 1984 (top) and 2012 (bottom).
